# Identification and Analysis of bZIP Family Genes in Potato and Their Potential Roles in Stress Responses

**DOI:** 10.3389/fpls.2021.637343

**Published:** 2021-05-28

**Authors:** Qi Wang, Cun Guo, Zhiyuan Li, Jinhao Sun, Dong Wang, Liangtao Xu, Xiaoxu Li, Yongfeng Guo

**Affiliations:** ^1^Tobacco Research Institute, Chinese Academy of Agricultural Sciences, Qingdao, China; ^2^Graduate School of Chinese Academy of Agricultural Sciences, Beijing, China; ^3^Technology Center, China Tobacco Hunan Industrial Co., Ltd., Changsha, China

**Keywords:** potato, bZIP, abiotic stress, transcription factor, gene family

## Abstract

The bZIP proteins comprise one of the largest transcription factor families and play important roles in plant growth and development, senescence, metabolic reactions, and stress responses. In this study, 49 bZIP transcription factor-encoding genes (*StbZIP* genes) on the potato genome were identified and analyzed. The 49 *StbZIP* genes, which are located on 12 chromosomes of the potato genome, were divided into 11 subgroups together with their *Arabidopsis* homologs based on the results of phylogenetic analysis. Gene structure and protein motif analysis revealed that members from the same subgroup often possessed similar exon/intron structures and motif organizations, further supporting the results of the phylogenetic analysis. Syntenic analysis indicated the existence of gene duplication events, which might play an important role in the expansion of the *bZIP* gene family in potato. Expressions of the *StbZIP* genes were analyzed in a variety of tissues *via* RNA-Seq data, suggesting functional diversity. Several *StbZIP* genes were found to be induced by different stress conditions. For example, the expression of *StbZIP25*, the close homolog of AtbZIP36/ABF2, was significantly upregulated by salt stress treatments. The StbZIP25 protein was found to be located in the nucleus and function as a transcriptional activator. Overexpression of *StbZIP25* enhanced salt tolerance in *Arabidopsis*. The results from this study imply potential roles of the bZIP family genes in the stress response of potato.

## Introduction

In the plant kingdom, transcription factors regulate various biological processes by activating or repressing gene expression (Qu and Zhu, 2006), among which the basic leucine zipper (bZIP) transcription factors have been reported to be one of the most diverse transcription factor families in plants, regulating various developmental processes and stress responses (Wang et al., 2015; Droge-Laser et al., 2018). The bZIP transcription factors possess a conserved bZIP domain, which is composed of 60–80 amino acids, including a DNA-binding basic region and a leucine (Leu) zipper domain (Landschulz et al., 1988; Droge-Laser et al., 2018). The basic region is highly conserved and consists of approximately 16 amino acid residues, containing an invariant N-X7-R/K-X9 motif that binds to specific DNA sequences with an ACGT core, such as A-box (TACGTA), C-box (GACGTC), and G-box (CACGTG) (Izawa et al., 1993; Niu et al., 1999). The Leu zipper domain is less conserved and consists of heptad repeats of leucine or other hydrophobic amino acids, which play an important role in specific recognition and dimerization (Jakoby et al., 2002; Wei et al., 2012; Wang et al., 2015).

In the model plant *Arabidopsis*, the bZIP family members have been classified into 13 groups, and most of them have been verified to have specific functions in regulating plant development (Droge-Laser et al., 2018). In group A, AtbZIP12/EEL and AtbZIP39/ABI5 regulate gene expression through competing for the same binding sites within the promoter of the *AtEm1* gene during late embryogenesis (Bensmihen et al., 2002). AtbZIP14/FD can form a complex with FLOWERING LOCUS T (FT) in controlling flowering by activating the floral identity gene *APETALA1* (*AP1*) (Wigge et al., 2005). In group C, AtbZIP9 was reported to be involved in vascular development in roots (Silveira et al., 2007). In group I, AtbZIP18 is a pollen-expressed bZIP transcription factor exhibiting functional redundancy with AtbZIP34 in male gametophyte development (Gibalova et al., 2017). AtbZIP29 was reported to function in leaf and root development (van Leene et al., 2016). AtbZIP30/DKM from group I negatively regulates *Arabidopsis* growth and reproductive development (Lozano Sotomayor et al., 2016). The size of *dkm* mutants was larger than that of wild-type individuals, with more floral buds, while compared to wild-type plants, *DKM* overexpression plants displayed a reduction in floral bud number (Lozano Sotomayor et al., 2016). AtbZIP44 in group S was reported to participate in regulating seed germination (Iglesias Fernandez et al., 2013).

Leaf senescence is a programmed developmental process and largely affects the yield and nutritional value of food crops (Gan and Amasino, 1997; Guo and Gan, 2014). The detached leaves of the triple mutant (*abf2abf3abf4*) displayed a stay-green phenotype after abscisic acid (ABA) treatment, indicating that AtbZIP36/ABF2, AtbZIP37/ABF3, and AtbZIP38/ABF4 in group A positively regulate ABA-mediated leaf senescence in *Arabidopsis* (Gao et al., 2016). *AtbZIP41*/*GBF1* in group G was highly expressed during leaf senescence and was reported to regulate the onset of leaf senescence *via* reducing the expression of the *CAT2* gene (Smykowski et al., 2010). Besides, AtbZIP1 and AtbZIP53 in group Scan synergistically regulate dark-induced leaf senescence (Dietrich et al., 2011). bZIP members have also been reported to be involved in plant metabolism. For example, in *Arabidopsis*, AtbZIP56/HY5 from group H was reported to regulate the biosynthesis of anthocyanins by binding the promoters of some *MYB* genes or inducing the expressions of biosynthesis enzyme genes (Shin et al., 2013). Similarly, in tomato, the bZIP transcription factor HY5 was also found to be involved in CRY1a-induced anthocyanin biosynthesis (Liu et al., 2018).

Furthermore, several bZIP proteins were reported to function in response to abiotic/biotic stresses in *Arabidopsis* (Banerjee and Roychoudhury, 2017). In group A, *AtbZIP35*/*ABF1* was highly upregulated by cold stress (Roychoudhury and Paul, 2012). AtbZIP36/ABF2 was reported to be involved in abiotic stress responses by regulating the expressions of stress-regulated genes (Kim et al., 2010). AtbZIP17 in group B and AtbZIP24 in group F were also reported to enhance salt tolerance *via* regulating stress response genes (Liu et al., 2008; Yang et al., 2009). AtbZIP51/VIP1 from group I was found to regulate osmotic pressure by directly binding to the promoters of *CYP707A1*/*3* genes (Tsugama et al., 2012). In group S, the expression of *AtbZIP1* was significantly upregulated under salt, cold, and drought stresses, and transgenic *Arabidopsis* plants overexpressing *AtbZIP1* had an enhanced tolerance to salt and drought stresses, indicating that AtbZIP1 functions as a positive regulator in plants in response to abiotic stresses (Sun et al., 2012). AtbZIP62, on the other hand, was reported to serve as a negative regulator of salt stress (Rolly et al., 2020).

Potato is an important economic crop all over the world. Increasing the resistance of potato to biotic/abiotic stresses for yield increase has been a hot research topic. With the availability of the Solanaceae genome database, studies of a number of potato gene families have been reported (Muniz Garcia et al., 2012; Zhou et al., 2018), but our understanding of the potato *bZIP* gene family is very limited. In this study, we have identified 49 potato *bZIP* genes from the Solanaceae database. Comprehensive analyses on the gene structure, promoter, chromosome distribution, phylogenetic analysis, and expression patterns were conducted, the results of which suggested that the potato bZIP members may play various roles in potato development and in response to stresses.

## Materials and Methods

### Identification and Classification of *bZIP* Genes in Potato

The potato genome annotations (PGSC, release 3.4) were retrieved from the Sol Genomics Network (SGN)^1^. Two different methods were used to identify the *bZIP* genes in potato: (1) BLASTP search using the *Arabidopsis* bZIP protein sequences according to previous methods (Li et al., 2018) and (2) the hidden Markov model profiles of bZIP domains (PF00170, PF07716, and PF03131) downloaded from Pfam (El Gebali et al., 2019) and used in searching potato proteome sequences *via* the HMMER (Johnson et al., 2010) program with an *E*-value cutoff of 0.001. All outputted bZIP protein sequences from the two methods were confirmed to have the bZIP domains using both InterProScan (Quevillon et al., 2005) and SMART (Letunic and Bork, 2018) databases. Candidate sequences that do not contain bZIP domains were manually deleted, and the remaining sequences were named according to their chromosome locations. Subsequently, potato bZIP protein sequences were submitted to the online tool ProtParam (Wilkins et al., 1999) to predict amino acid quantity, molecular weight, and isoelectric point.

Phylogenetic analysis was performed using the previously reported *Arabidopsis* bZIP sequences (Droge-Laser et al., 2018) and the newly identified potato bZIP protein sequences. Multi-sequence alignment was performed using MAFFT (Katoh et al., 2019), and the result was presented with the Texshade program (Beitz, 2000). Based on the alignment results, MEGA was used to build a neighbor-joining (NJ) tree under the default parameters. The FigTree software was employed to visualize the tree file (University of Maryland, College Park, MD, United States).

### Exon–Intron Structural Analysis and Identification of Conserved Motifs

The Gene Structure Display Server (Hu et al., 2015) was used to visualize the exon–intron structures of the *Arabidopsis* and potato *bZIP* genes by submitting their genomic and coding sequences. The conserved motifs of their protein sequences were identified *via* Multiple Em for Motif Elicitation (MEME) tools (Bailey et al., 2009) with the following parameters: distribution of motif occurrences, zero or one per sequence; maximum number of motifs, 10; and the optimum width of each motif, between six and 100 residues.

### Chromosomal Localization and Duplication Event Analysis of Potato *bZIP* Genes

The chromosomal location information of potato *bZIP* genes were downloaded from the SGN database and displayed visually with Perl. Tandem gene events were defined as previously described (Li et al., 2018) and displayed on the potato chromosomes. For syntenic analysis, we searched the synteny relationship of the orthologous genes from potato and four other plant species (including *Arabidopsis*, tomato, grape, and rice), and the results were displayed with TBtools (Chen et al., 2020). Subsequently, the synonymous substitution (Ks) and non-synonymous substitution (Ka) rates were calculated using the DnaSP 5.0 software (Librado and Rozas, 2009).

### Analysis of *cis-*Elements in the Promoter of Potato *bZIP* Genes

To assess the promoter *cis*-acting elements of the *StbZIP* genes, 2,000 bp of promoter regions upstream of the start codon of the *StbZIP* genes were extracted. PlantCARE^2^ was engaged for *cis*-acting regulatory element investigation.

### Expression Pattern Analysis of Potato *bZIP* Genes

To determine the expressions of *StbZIP* genes in different tissues, RNA sequencing (RNA-Seq) data were obtained from the PGSC database (Xu et al., 2011). The expression data of the root, stem, shoot apex, leaf, flower, and tuber were selected. The expression data of biotic and abiotic stresses were also downloaded and the relative expression calculated relative to their controls, respectively. Only genes with absolute expression values of log multiples > 2 were displayed. The resulted data were normalized and illustrated by R. This R package has been integrated in TBtools (Chen et al., 2020).

### Potato Plant Preparation and Salt Stress Treatments

The potato cultivar GN2 (Gannongshu 2) was used to analyze the expressions of *StbZIP* genes. Potato sprouts were incubated on complete Murashige and Skoog (MS) solid medium by nodule cutting and cultivated in a growth chamber at 24°C under continuous light (70 ± 5 μmol m^–2^ s^–1^ photosynthetic photon flux density) and 50–60% relative humidity. For salt stress treatments, 4-week-old seedlings were treated with 150 mM NaCl. After treatments of 0, 1, 3, and 6 h, whole plants were harvested for RNA extraction and real-time PCR (RT-PCR) analysis. The harvested samples were immediately frozen in liquid nitrogen and stored at −80°C prior to RNA extraction. Three biological replicates were used for each sample.

### RNA Extraction and Real-Time Polymerase Chain Reaction Analysis

Total RNAs were extracted using the Ultrapure RNA Kit (cwbiotech, Beijing, China), and the first-strand complementary DNA (cDNA) was synthesized using the PrimeScript^TM^ RT reagent Kit (TaKaRa). The potato *EF1*α gene was adopted as an internal control (Singh et al., 2013), and quantitative RT-PCR (qRT-PCR) reactions were performed with 40 cycles in a Roche LightCycler 480 Real-Time PCR instrument. All expression data were obtained from three technical repeats and calculated by the 2^–ΔΔ*CT*^ method. Statistical significance was analyzed using SPSS v18.0 *t* test. The primer sequences used in the current study are listed in Supplementary Table 1.

### Subcellular Localization and Transactivation Assays

The *StbZIP25* coding sequence (CDS) without stop codon was PCR amplified from potato cDNAs and cloned into the pEasy-Blunt subcloning vector then inserted into the PYG57 vector, which contains the green fluorescent protein (GFP) coding sequence, to construct the *StbZIP25*–*GFP* fusion gene driven by the CaMV-35S promoter. The construct was then transformed into *Agrobacterium* GV3101 competent cells and transiently expressed in the leaves of *Nicotiana benthamiana*. Simultaneously, the empty PYG57 vector was used as a control. Three days after injection, the leaves were soaked in the DAPI staining solution and observed under a confocal laser microscope (TCS-SP8 Leica, Wetzlar, Germany) to detect fluorescence signals of the fusion protein, as previously reported (Li et al., 2019a, b).

For the transactivation activity assay of StbZIP25, the full-length CDS of the *StbZIP25* gene was PCR amplified and inserted into the pBridge vector at the *Eco*RI site by Infusion (Clontech, Bejing, China) to fuse with a GAL4 DNA binding domain, yielding plasmid pBridge-StbZIP25. Plasmid pBridge-StbZIP25 and the empty pBridge vector (control) were then transformed into the yeast strain AH109, and the transformed yeasts were plated on synthetic dextrose (SD) media lacking tryptophan (SD/-Trp) and incubated at 30°C for 3 days. Subsequently, the positive clones were transferred to SD/-Trp media supplemented with X-gal and incubated at 30°C for 3 days. The transactivation activity of the fused proteins was determined based on the growth status (blue/white) of the transformants.

### Overexpression Analysis

The *StbZIP25* gene coding sequence was PCR amplified and inserted into the pCHF3 vector to generate the *StbZIP25* overexpression construct driven by the CaMV-35S promoter, which was then transformed into *Arabidopsis* wild-type (Col-0) plants by the floral dip method (Zhang et al., 2006). The T0 generation seeds were screened on half-strength MS medium with 50 mg/L kanamycin to obtain *StbZIP25* overexpression plants. Seven-day-old T3 transgenic and wild-type (WT) *Arabidopsis* plants were transferred to 1/2 MS media with or without NaCl (100 mM) to be grown vertically in a growth chamber (23°C, continuous light). The primary root length of 18 plants was measured after 21 days. Significant difference analysis was calculated by SPSS v18.0 with the *t* test program.

## Results

### Identification of *StbZIP* Genes

The BLASTp method was used to identify *bZIP* genes from the potato genome. A total of 49 *StbZIP* genes were identified after determining the presence of a complete bZIP motif in each candidate. The 49 *StbZIP* genes were distributed on 12 potato chromosomes and were named from *StbZIP01* to *StbZIP49*, according to their chromosomal locations.

Characteristics of the StbZIP proteins including the number of amino acids, molecular weight (*M*_*w*_), and the isoelectric point (p*I*) were analyzed (Supplementary Table 2). The length of the 49 StbZIP proteins ranged from 132 (StbZIP12) to 822 (StbZIP46) amino acids. The molecular weight of the bZIP proteins ranged from 15.41 (StbZIP04 and StbZIP05) to 88.18 (StbZIP46) kDa, and p*I* ranged from 5.02 (StbZIP35) to 10.31 (StbZIP07).

### Multiple Sequence Alignment and Phylogenetic Analysis

In plants, the bZIP proteins possess a highly conserved DNA-binding basic region. To investigate the characteristics of the potato bZIP members, full-length sequences of all the newly identified StbZIP proteins were subjected to multiple sequence alignments, and the basic region was visualized by R. The basic region possesses a nuclear localization signal and an invariant N-X7-R/K-X9 motif (Supplementary Figure 1A). In addition, the characteristics of the basic region sequence were highly conserved between potato and *Arabidopsis* (Supplementary Figures 1A,B).

**FIGURE 1:**
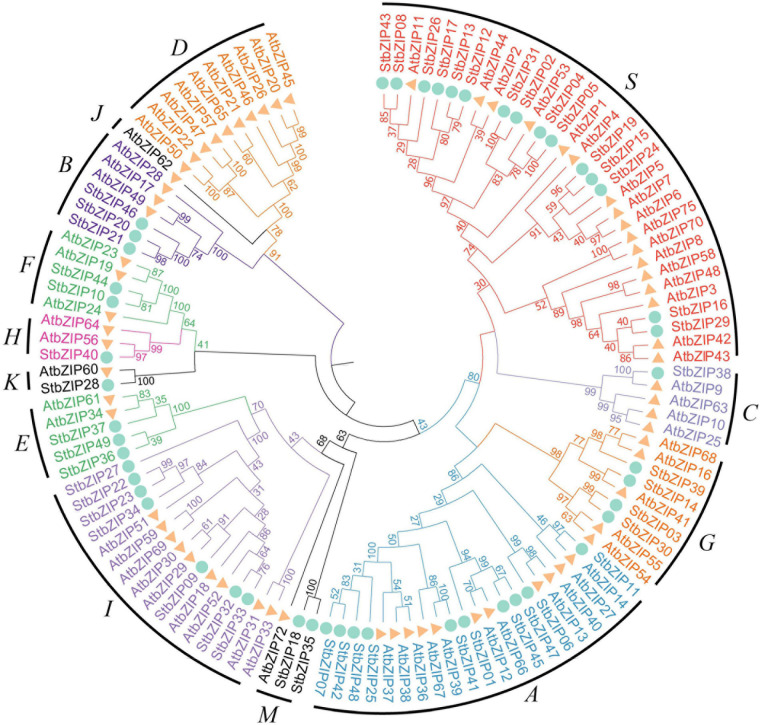
Phylogenetic analysis of potato bZIP family members. The phylogenetic tree was generated from the alignment of potato and *Arabidopsis* bZIP proteins with 1,000 bootstrap replicates using the neighbor-joining (NJ) method. The potato bZIP members together with their *Arabidopsis* homologs were classified into 11 groups.

To explore the phylogenetic relationship of the StbZIP proteins, a neighbor-joining tree was constructed based on multiple sequence alignment of the 49 potato bZIP members and their *Arabidopsis* homologs (Figure 1). Based on the results of the phylogenetic analysis and the results of a previous study in which *Arabidopsis* bZIP members were classified into 13 groups (Droge-Laser et al., 2018), the 49 potato bZIP proteins were classified into 11 groups together with their *Arabidopsis* homologs. Notably, most of the groups contained two or more potato bZIP members, indicating that the differentiation time of potato and *Arabidopsis* was later than that of the *bZIP* gene family. Among the groups, group S contained the largest number of members (15), while groups C and H contained the minimum number of members (one). Intriguingly, it was found that only the *Arabidopsis* bZIP proteins fall into groups D and J, indicating that the *StbZIP* genes in these groups might have been lost during the evolution of potato.

### *StbZIP* Genes Syntenic Analysis

**FIGURE 2:**
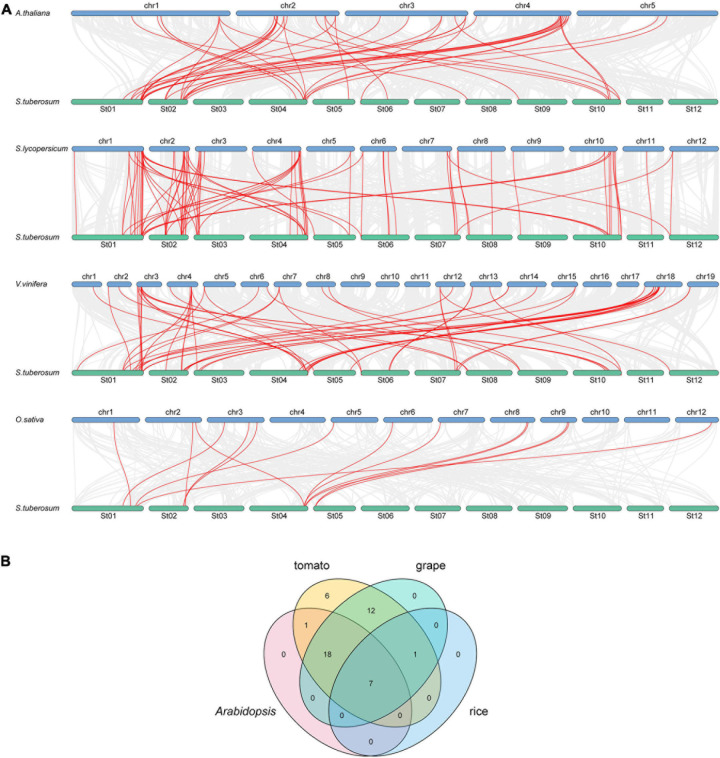
Synteny analysis of *bZIP* genes between potato and four other representative plant species. **(A)** The gray line in the background represents the collinear blocks between potato and four other representative species, while the red line exhibit the syntenic *bZIP* gene pairs. **(B)** The numbers of *bZIP* genes that formed syntenic pairs between potato and all the other four selected species which visualized by the Venn plot.

To further study the evolutionary relationship among the potato *bZIP* genes, syntenic analysis was carried out for potato and four other plant species, including three dicots (*Arabidopsis*, tomato, and grape) and one monocot (rice) (Figure 2A). The results showed that there was a syntenic relationship between 45 of the *StbZIP* genes with the *bZIP* genes in tomato, 38 *StbZIP* genes in grape, 26 *StbZIP* genes in *Arabidopsis*, and eight *StbZIP* genes in rice. The numbers of predicted collinear pairs between potato and tomato, grape, *Arabidopsis*, and rice, were 81, 56, 44, and 14, respectively.

Seven *StbZIP* genes were predicted to form collinear pairs with the *bZIP* genes of all the other four species, which indicates that these genes may have existed before the differentiation of these species and have maintained a collinear relationship since then. It is worth noting that a total of 18 collinear gene pairs were identified between potato and tomato/grape/*Arabidopsis* species, but no collinear gene pair was found in the rice genome, indicating that those pairs may have appeared after the divergence of dicots and monocots. Interestingly, 12 *AtbZIP* genes were predicted to have a collinearity relationship with two or more *StbZIP* genes, which implies that the formation of these *StbZIP* genes may be caused by duplications and might have played significant roles in the evolution of potato (Figure 2B and Supplementary Table 3).

### Gene Structure and Motif Composition

As shown in Supplementary Figure 2, the gene structures of potato and the *Arabidopsis bZIP* genes were drawn with the online software GSDS and displayed visually. The numbers of introns of these genes range from zero to 11. Among them, 15 potato *bZIP* genes have no introns. Interestingly, all the genes without intron were classified into group S. In addition, the genes with the largest number of introns were distributed in group G, and the average number of introns in this group is 10. The genes of groups A and I all contain two to three introns, while group C genes contain five to six introns. *bZIP* genes of the same group have similar gene structures, indicating that the above-described evolutionary relationship and classification analysis of *StbZIP* genes were reliable. It is worth mentioning that, although the average number of introns in *Arabidopsis* (10.6) and potato (10.5) is similar, group D, which only contains *Arabidopsis* genes, shows the largest variation in gene structure, with the number of introns ranging from 6 to 11.

Subsequently, the MEME online tool was used to analyze the conserved domains of potato and *Arabidopsis* bZIP proteins. A total of 10 conserved functional domains were identified (Supplementary Table 4). Among them, motif 1 represents the basic leucine zipper domain, and all the identified bZIP members harbor motif 1 (Supplementary Figure 2). Members of the same group share similar motifs. The members of groups G, H, F, and B only contain motif 1, members of group S contain motifs 1 and 5, group I and group E members harbor motifs 1 and 2, and members of group A harbor motifs 1, 6, 7, 8, and 9. It is worth noting that motifs 3, 4, 9, and 10 were highly conserved and only existed in group D, indicating that they were conserved protein domains which may undertake a special function.

### Chromosomal Distribution and Duplication Events

**FIGURE 3:**
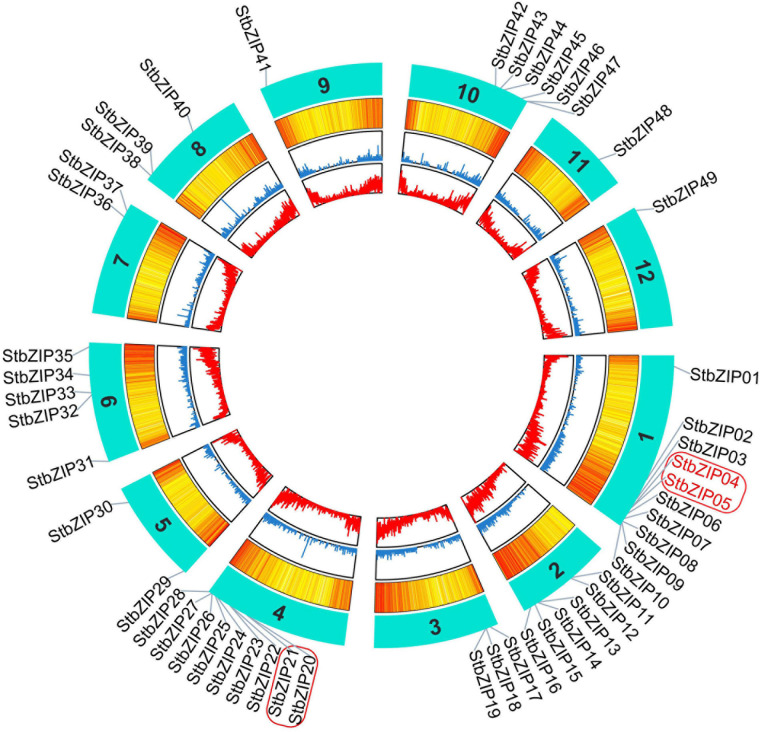
Distribution of StbZIP genes on potato chromosomes. In potato, 49 StbZIP genes were successfully mapped to 12 potato chromosomes. The red box indicate the gene cluster, while the tandem duplication pair is featured by the red color.

The 49 *StbZIP* genes were mapped onto 12 potato chromosomes (Figure 3). The most potato *bZIP* genes (10) were found on chromosome 1, while chromosomes 9, 11, and 12 only harbor one potato *bZIP* gene. It has been reported that when a chromosome region within 200 kb possesses two or more genes of the same family, these genes are defined to be a gene cluster, and genes sharing an identity of more than 70% in a cluster are considered to be tandem duplication genes (Holub, 2001). Homology analysis showed that there is one cluster of *bZIP* genes on chromosome 1 and chromosome 4. In addition, there are two *StbZIP* genes (*StbZIP04*/*StbZIP05*) that formed a tandem duplication pair.

**FIGURE 4:**
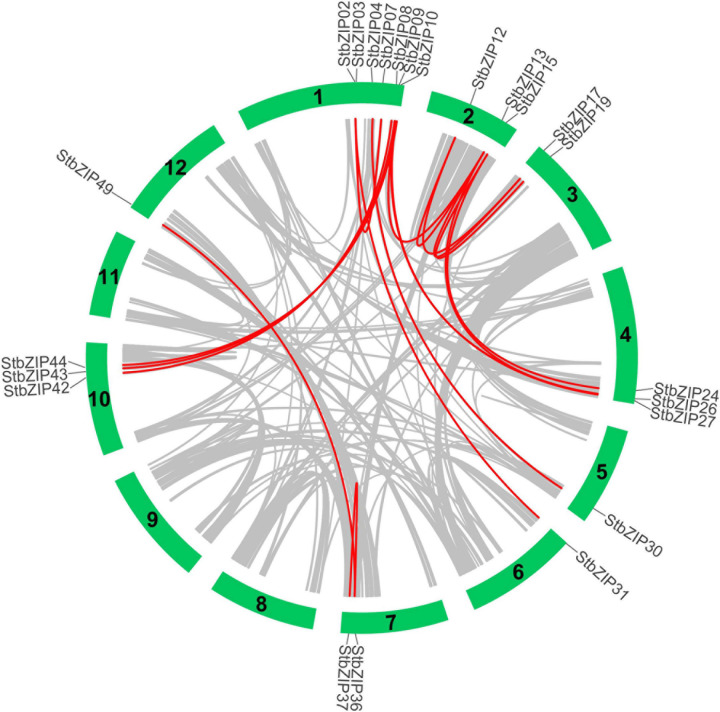
Segmental duplication events and interchromosomal relationships between StbZIP genes. The 31 putative segmental duplication pairs of StbZIP genes were investigated with MCScanX and linked by the colored lines, respectively. The gray lines indicate all putative segmental duplication pairs in the potato genome, while the StbZIP segmental duplication pairs are linked by the red line.

Furthermore, MCScanX was used to analyze segmental duplication or whole-genome duplication events of the potato *bZIP* genes. A total of 17 pairs of segmental duplication with 23 *StbZIP* genes were identified (Figure 4). These results showed that the formation of some *StbZIP* genes may be caused by duplication events and that these segmental duplication events might play a major role in the evolution of *StbZIP* genes. All of the tandem and segmental duplication genes are listed in Supplementary Table 5.

The ratio of Ka/Ks and the ratio between non-synonymous and synonymous substitutions can be used to estimate whether the selective pressure acts on a protein-coding gene. There are three selection types in evolutionary analysis, including positive selection, neutral selection, and purifying selection. All the Ka/Ks ratios of the 17 segmental duplication pairs were lower than 1, suggesting that these *bZIP* genes may have undergone purifying selective pressure in the process of evolution.

### Promoter Analysis of *StbZIP* Genes

**FIGURE 5:**
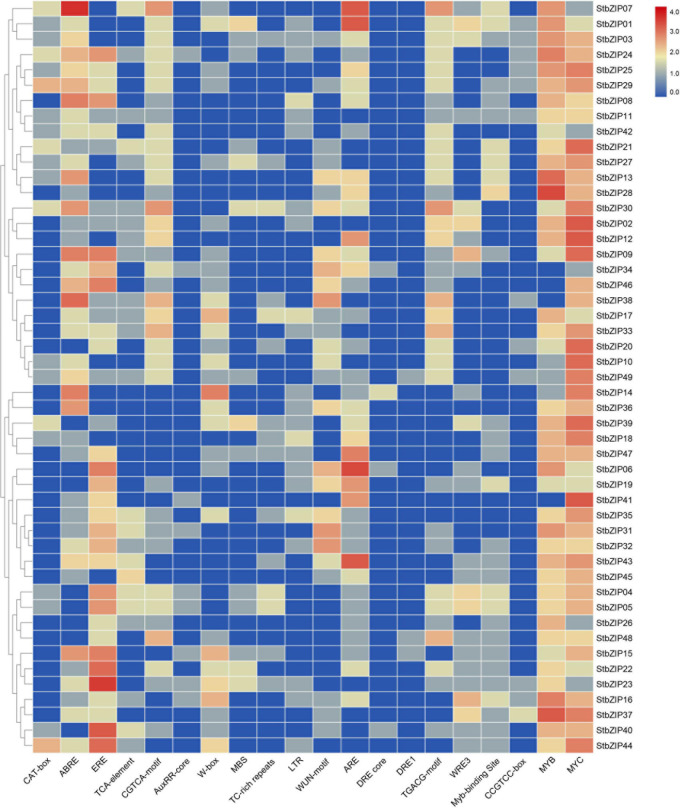
Regulatory elements in the promoter regions of *StbZIP* genes.

Several putative *cis*-elements on promoter regions were identified in the 49 *StbZIP* gene promoters (Figure 5 and Supplementary Table 6). One or more MYC elements were identified in the promoters of all the *StbZIP* genes. In addition, hormone-responsive elements, including ABRE, ERE, and the CGTCA motif, were identified in the promoter regions of some *StbZIP* genes. These *cis*-elements are potentially responsive to abscisic acid, ethylene, and methyl jasmonate. Furthermore, 37 of the *StbZIP* genes contain an anaerobic induction element (ARE), 29 contain at least one WRKY binding site, 27 contain at least one wound-responsive element (WUN motif), 22 contain at least one low-temperature-responsive element (LTR), and 15 of the genes contain at least one stress-responsive element (TC-rich repeats), implying that these *StbZIP* genes may be involved in various stress responses.

### Expression of *StbZIP* Genes in Different Tissues

**FIGURE 6:**
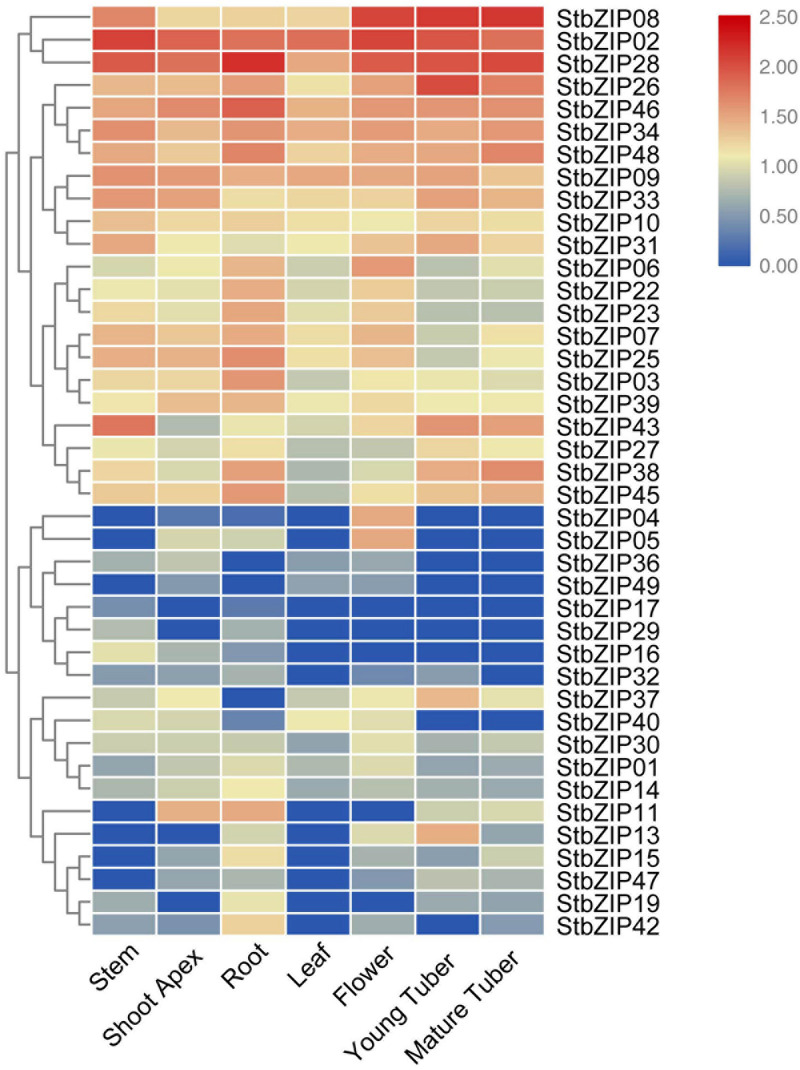
Expression patterns of the *StbZIP* genes in the tested tissues. The expression pattern data were retrieved from transcriptome data and visualized by TBtools.

To understand the potential functions of the *StbZIP* genes, their tissue-specific expression levels were analyzed using RNA-Seq data from the PGSC database. Seven tissues were selected for analysis, including the root, stem, shoot apex, leaf, flower, young tuber, and mature tuber. Forty-one of the 49 *StbZIP* genes were expressed in at least one tested tissue, and the transcriptome data were standardized and displayed in R (Figure 6). A number of *StbZIP* genes were expressed in all the tested tissues, including *StbZIP08*, *StbZIP02*, *StbZIP26*, *StbZIP31*, and *StbZIP43* from group S. Other *StbZIP* genes showed tissue-specific expression patterns. The expression of *StbZIP04* was only detected in the flower, *StbZIP42* was expressed exclusively in the root, and *StbZIP17* and *StbZIP29* were only expressed in the roots and stems. Furthermore, compared with the other tissues, *StbZIP13* and *StbZIP37* were specifically upregulated in young tubers.

### Expression Patterns of *StbZIP* Genes in Response to Stress Treatments

**FIGURE 7:**
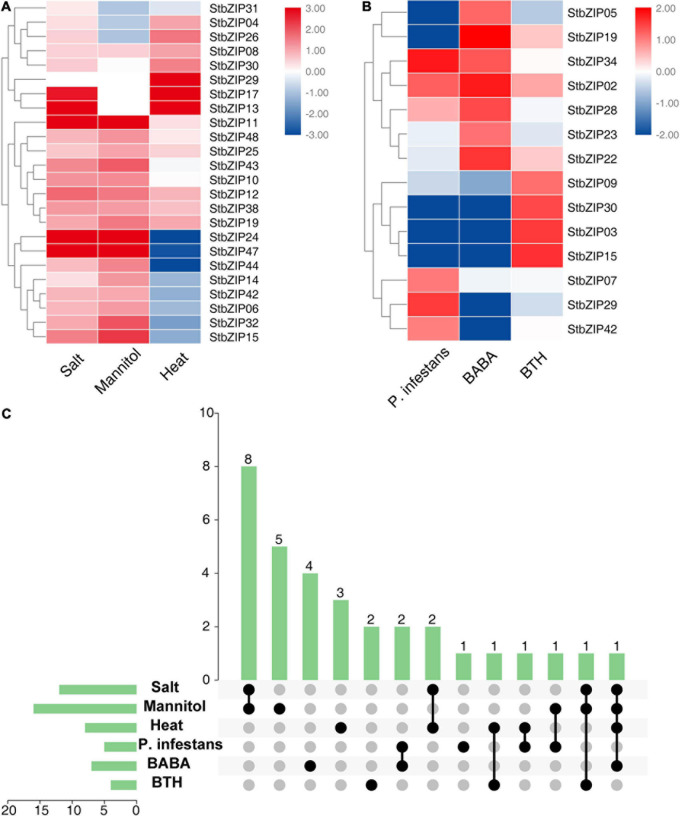
The expression patterns of *StbZIP* genes under abiotic and biotic stress treatments. **(A)** Relative expression ratios of the abiotic stress treatments. **(B)** Relative expression ratios of the biotic stress treatments. **(C)** Summarized information of the stress-induced *StbZIP* genes. The relative expression ratios of the abiotic and biotic stress treatments were calculated relative to the untreated control and then the significantly induced gene was defined to possess a log2 relative expression ratio ≥ 1 under one of the stress treatments. The red, white, and blue colors represent the upregulated, unaltered, and downregulated expressions, respectively. The vertical axis and *vertical bars* represent the number of genes responding to stresses. The points represent the number of genes responding to one or more stresses.

The *bZIP* genes have been previously reported to be involved in biotic and abiotic stresses (Banerjee and Roychoudhury, 2017). To investigate the roles of *StbZIP* genes under stresses, their gene expression changes under biotic and abiotic stresses were analyzed. Salt, mannitol, and heat treatments were used for abiotic stresses, and *Phytophthora infestans*, β-aminobutyric acid (BABA), and benzothiadiazole (BTH) treatments were employed for biotic stresses. The results indicated that 24 *StbZIP* genes responded to abiotic stresses (Figure 7A), among which *StbZIP25* was induced by salt, mannitol, and heat treatments; *StbZIP29* was only highly expressed under heat stress; *StbZIP13* and *StbZIP17* were highly expressed under salt and heat stresses; and *StbZIP11*, *StbZIP24*, and *StbZIP47* were significantly induced under salt and drought stresses. Interestingly, the expressions of *StbZIP24* and *StbZIP47* were suppressed by heat stress. The expression patterns of *StbZIP44*, *StbZIP14*, *StbZIP42*, *StbZIP06*, *StbZIP32*, *StbZIP15*, *StbZIP24*, and *StbZIP47* were similar in response to stresses: induced by salt and drought, while suppressed by heat.

Under biotic stresses, 14 *StbZIP* genes presented differential expressions when compared with the untreated control (Figure 7B). Among these, only *StbZIP02* was upregulated after all three biotic stresses. *StbZIP34* and *StbZIP28* were found to be upregulated under the stress of BABA. On the contrary, the expressions of *StbZIP30*, *StbZIP03*, and *StbZIP15* were decreased under BABA stress. *StbZIP29* was upregulated after *P. infestans* inoculation, while *StbZIP05* and *StbZIP19* exhibited downregulated expressions under this stress.

Furthermore, we found that 32 *StbZIP* genes were induced under both abiotic and biotic stress conditions (Figure 7C). *StbZIP29* was highly induced by *P. infestans* inoculation and under heat stress treatments, and *StbZIP15* was upregulated by BTH and in response to salt and drought treatments. Similarly, *StbZIP19* responded to all abiotic stresses and BABA treatment (Supplementary Table 7).

### Validation of Expression Patterns by qRT-PCR

**FIGURE 8:**
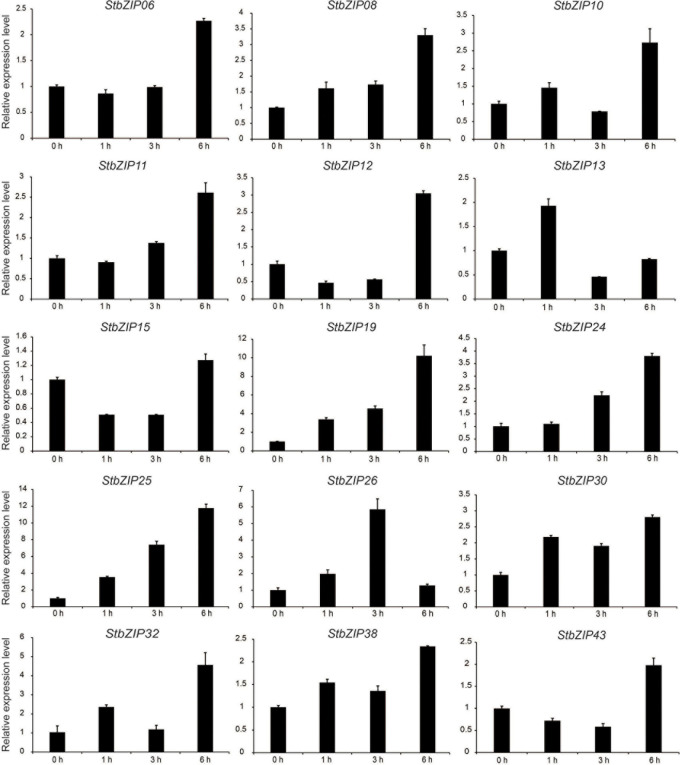
The expression patterns of selected *StbZIP* genes in response to salt stress treatments, which were calculated as folds relative to the untreated control.

qRT-PCR was conducted to verify the RNA-Seq expression patterns of potato *bZIP* genes under salt stress. As shown in Figure 8, the expression levels of the selected 15 *bZIP* genes were all enhanced by the salt treatment, which was consistent with the RNA-Seq data. Among them, the expression levels of eight *StbZIP* genes kept increasing with the increase of salt treatment time. This included *StZIP06*, *StZIP08*, *StZIP10*, *StZIP11*, *StZIP12*, *StZIP19*, *StZIP24*, and *StZIP25*. It is worth noting that StbZIP10 is a close homolog of AtbZIP24, which was reported to enhance salt tolerance *via* regulating stress-responsive genes (Yang et al., 2009), suggesting that StbZIP10 may also be involved in potato salt stress response. The expression patterns of the other genes were somewhat complicated. For instance, *StZIP13* and *StZIP32* were highly induced after 1 h of salt treatment, then decreased after 3 h, and finally increased again after 6 h treatment. *StZIP26* expression was in a similar up–down–up pattern, except that it reached the peak after 3 h of treatment then began to decline. The expressions of *StZIP15* and *StZIP43* were both suppressed after 1 h of salt treatment, then increased 6 h after treatment, suggesting that there might be feedback activation mechanisms involved in their response to salt stress treatments.

### Subcellular Location and Transactivation Activity Assays

**FIGURE 9:**
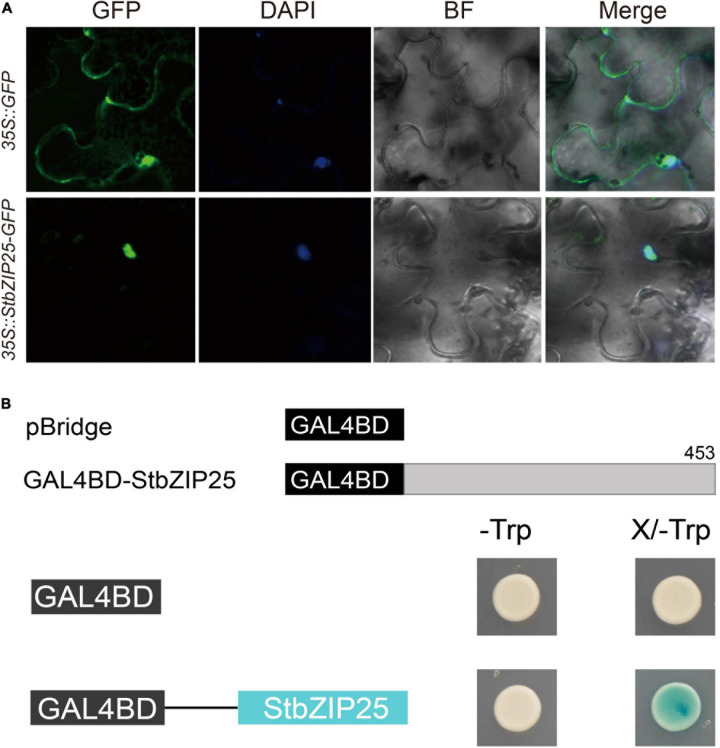
The subcellular localization and transactivation assays of StbZIP25. **(A)** The *StbZIP25-GFP* fusion construct and the *GFP* gene driven by the CaMV-35S promoter were transiently expressed into tobacco. DAPI (dye 4,6-diamidino-2-phenylindole) staining indicated the nucleus. **(B)** Transactivation activity assay of StbZIP25 in yeast strain AH109. β-galactosidase activities against X-gal were detected on SD/-Trp media.

In order to analyze the subcellular localization of the StbZIP proteins, the group A member StbZIP25 was selected as a representative to perform the experiment. The *35S:StbZIP25-GFP* recombinant construct and the *35S:GFP* control were transiently expressed in *N. benthamiana* and the subcellular localization of the GFP signal was observed by confocal microscopy. As a result, the green fluorescence of the *35S:GFP* control in tobacco leaf epidermal cells was distributed on plasma membranes and in cytoplasm, whereas the StbZIP25–GFP fusion proteins were only found in the nucleus, indicating that the StbZIP25 protein was localized in the nucleus (Figure 9A).

To investigate whether StbZIP25 acts as a transcriptional activator, transcriptional activation assays were performed using the StbZIP25 full-length CDS (Figure 9B). Transformed AH109 yeast cells selected on SD/-Trp media were transferred to SD/-Trp/X-gal media. Activation of the reporter gene in the yeast cells was determined by assays of the β-galactosidase activities with X-gal as the substrate. The yeast cells containing the full-length StbZIP25 (GAL4BD-StbZIP25) were blue on the SD/-Trp/X-gal media, whereas the cells with the pBridge empty vector was white (Figure 9B), indicating that StbZIP25 functioned as a transcriptional activator.

### Overexpression of *StbZIP25* Enhanced Salt Tolerance in *Arabidopsis*

**FIGURE 10:**
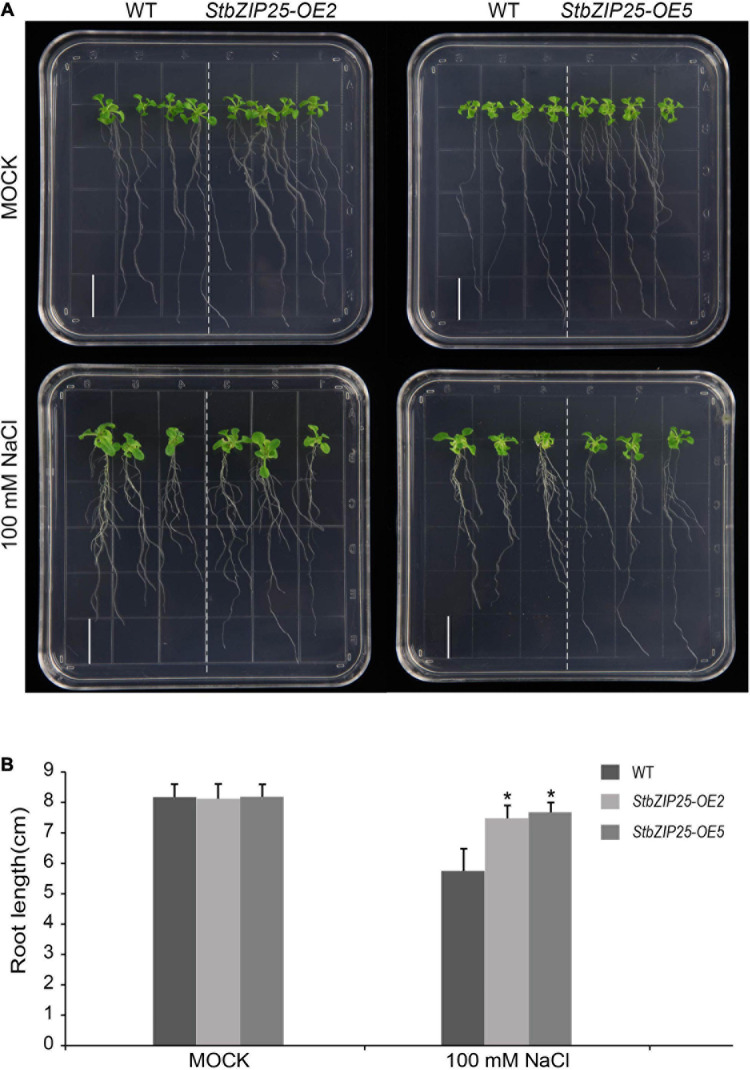
Effects of salt stress treatments on the root growth of the *StbZIP25* gene overexpressed in *Arabidopsis*. **(A)** Primary root lengths of the wild-type and *StbZIP25* overexpression lines under salt treatments in transgenic *Arabidopsis*. **(B)** Quantification of the primary root lengths under normal condition and 100 mM NaCl treatments. *Bar*, 1.5 cm. The data were retrieved from three biological replicates. WT, wild type. Data are the mean ± SD of three biological repeats. *p < 0.05 (*t* tests).

To further explore the function of the salt-responsive gene *StbZIP25* in abiotic stresses, transgenic *Arabidopsis* plants overexpressing *StbZIP25* were generated. Five T0 transgenic lines were verified by PCR using genomic DAN templates. The homozygous T3 transgenic lines were selected and the expression level of *StbZIP25* was analyzed using qRT-PCR. Two overexpression lines (*OE2* and *OE5*) with higher expression levels of *StbZIP25* were selected for further analysis (Supplementary Figure 3). The root length of plants grown on 1/2 MS medium with 100 mM NaCl was measured to assess salt tolerance (Figure 10). WT and *StbZIP25*-overexpressing *Arabidopsis* plants showed no significant difference in root length under normal conditions. However, after 3 weeks of growing on 100 mM NaCl media, the transgenic plants displayed a longer root phenotype compared with the WT. Taken together, the results revealed that the overexpression of *StbZIP25* enhanced the salt tolerance in transgenic *Arabidopsis*.

## Discussion

The *bZIP* gene family has been reported to be one of the largest transcription factor families in plants that functions in plant development, metabolism, and stress responses (Wang et al., 2015; Droge-Laser et al., 2018). Furthermore, bZIP proteins have been identified in a number of plant species, including *Arabidopsis* (Jakoby et al., 2002), maize (Wei et al., 2012), rice (Nijhawan et al., 2008), sorghum (Wang et al., 2011), and wheat (Li et al., 2015). In this study, a total of 49 *bZIP* genes were identified from the potato genome by using BLASTP searches. The number of *StbZIP* genes was lower than the number of *bZIP* genes in *Arabidopsis* (75 genes) (Jakoby et al., 2002) and was similar to that in grapevine (55 genes) (Liu et al., 2014) and in castor bean (49 genes) (Jin et al., 2014). The potential potato *bZIP* genes were studied through analyses of the phylogeny, gene structure, motif organization, synteny, chromosomal distribution, duplication events, *cis*-elements, and expression profiles.

Tandem and segmental duplication play key roles in the extension of a gene family (Vision et al., 2000; Cannon et al., 2004). In this study, 17 segmental duplication pairs were found in the potato *bZIP* gene family, whereas only one tandem duplication pair was identified (Figures 3, 4), suggesting that the expansion of the *StbZIP* gene family in potato was mainly originated from segmental duplication. The Ka/Ks ratios of all these duplicated pairs were lower than 1 (Supplementary Table 5), suggesting that these duplicated *StbZIP* genes might have been purifying selected and maintained conserved functions in evolution. For example, *StbZIP04* and *StbZIP05*, as a tandem duplication pair, were both highly expressed in flower tissues (Figure 6).

A number of *bZIP* genes have been reported to be involved in controlling *Arabidopsis* development. In group C, AtbZIP9/BZO2H2 has been reported to alter vascular development in roots (Silveira et al., 2007). *StbZIP38* was clustered together with AtbZIP9 in group C, and this gene showed a high level of expression in roots (Figure 6), implying that StbZIP38 may also be involved in root development. In group I, AtbZIP29 was reported to function in leaf and root development (van Leene et al., 2016). Its homolog, *StbZIP09*, was found to be highly expressed in both roots and leaves, which suggested that AtbZIP29 and StbZIP09 might have similar biological functions. AtbZIP44 in group S was reported to participate in regulating seed germination (Iglesias Fernandez et al., 2013). Although gene expression in seeds was not examined in this study, a close homolog of AtZIP44 in potato, *StbZIP08*, was found to be highly expressed in the flower (Figure 6).

Increasing evidence indicates that bZIP family members can function as regulators in plants’ responses to abiotic stresses. AtbZIP24 in group F, which has been reported to be involved in regulating abiotic stress responses (Yang et al., 2009), was clustered together with StbZIP10 and StbZIP44. Interestingly, *StbZIP10* and *StbZIP44* were found to be induced by salt/drought stresses (Figure 7A). In addition, the ABF subfamily members of bZIP proteins are involved in ABA/stress responses (Banerjee and Roychoudhury, 2017). The overexpression of *AtbZIP36*/*ABF2* altered ABA sensitivity, dehydration tolerance, and the expression levels of ABA/stress-regulated genes (Kim et al., 2010). The promoter of the AtbZIP36/ABF2 homologous gene *StbZIP25* contains the *cis*-element ABRE (Figure 5), suggesting that it may also be related to ABA signaling and stress responses. In addition, salt and drought stress treatments can significantly induce the expression of *StbZIP25* (Figures 7A, 8), suggesting that it may participate in the response to plant salt and drought stresses. Further experiments indicated that the overexpression of *StbZIP25* can confer salt tolerance in transgenic *Arabidopsis* (Figure 10), supporting the role of StbZIP in stress response and the potential functional conservation of plant *bZIP* genes between species. The StbZIP25 protein was shown to be located in the nucleus and showed transcriptional activation activities (Figure 9), indicating that StbZIP25 acts as a transcription activator to regulate gene expression in response to stresses. StbZIP25 was chosen as a representative of StbZIP members showing stress-responsive expressions which could potentially function as regulators of stress responses. The results of *StbZIP25* overexpression conferred salt tolerance in *Arabidopsis* (Figure 10), supporting the potential roles of more StbZIP proteins in stress responses.

## Conclusion

In this study, a systematic study of the potato *bZIP* gene family was carried out, including the identification of the potato bZIP family members, analyses on evolutionary relationship, chromosome locations, and the expression patterns of *StbZIP* genes. The results suggest that the *StbZIP* genes may be important for regulating plant responses to abiotic/biotic stresses and development. Notably, AtbZIP36/ABF2 has been reported to participate in regulating the response to abiotic stresses. Its potato homolog *StbZIP25* was induced by salt and drought stresses and was able to enhance salt tolerance in transgenic *Arabidopsis*. Overall, the results from this study will help in further investigations of the function of potato *bZIP* genes.

## Data Availability Statement

The original contributions presented in the study are included in the article/Supplementary Material, further inquiries can be directed to the corresponding authors.

## Author Contributions

XL and YG conceived this research, designed the experiments, and drafted the manuscript. QW and CG conducted the research and participated in drafting the manuscript. ZL, JS, DW, and LX assisted in data collection and analysis. All authors contributed to the article and approved the submitted version.

## Conflict of Interest

XL, DW, and LX were employed by China Tobacco Hunan Industrial Co., Ltd. The remaining authors declare that the research was conducted in the absence of any commercial or financial relationships that could be construed as a potential conflict of interest.

## Footnotes

1 http://solgenomics.net/

2 http://bioinformatics.psb.ugent.be/webtools/plantcare/html/

## Supplementary Material

Supplementary Figure 1 Sequence logos of the basic region of bZIP members from potato **(A)** and *Arabidopsis* **(B)**.

Supplementary Figure 2 The motif and gene structure organizations of StbZIP members.

Supplementary Figure 3 **(A)** Five T0 transgenic lines were verified by normal PCR using genomic DNA templates. **(B)** The expression levels of *StbZIP25* gene in wild type and two overexpression lines. The ratios of gene expression were calculated relative to the wild type.

Supplementary Table 1 The qRT-PCR primers used in this study.

Supplementary Table 2 The detail information of identified potato bZIP family members.

Supplementary Table 3 The syntenic pairs between potato and other four plant species.

Supplementary Table 4 The putative conserved motifs in StbZIP proteins.

Supplementary Table 5 The detail information of segmental and tandem duplication gene pairs.

Supplementary Table 6 Regulatory elements in the promoter regions of StbZIP genes.

Supplementary Table 7 The detail information of the induced *StbZIP* gene under various stress treatments.
