# Supplementary figures and images for: Identification and Analysis of bZIP Family Genes in Potato and Their Potential Roles in Stress Responses
Source: Front Plant Sci. 2021 May 28;12:637343. doi: 10.3389/fpls.2021.637343 (PMC8193719; doi:10.3389/fpls.2021.637343)

A

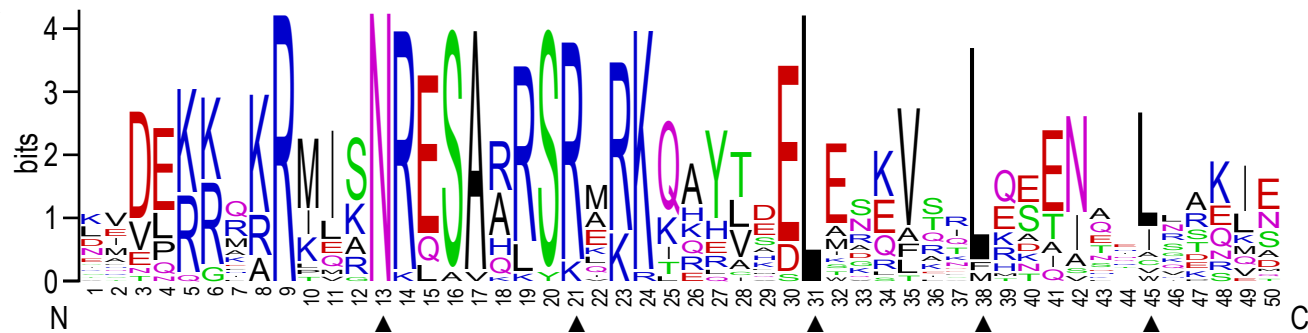

B

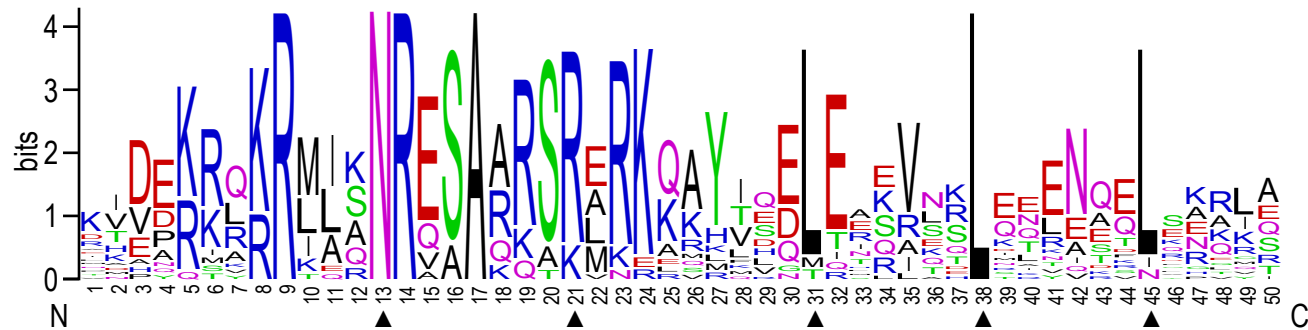

Supplement: Supplementary Figure 1 — Sequence logos of the basic region of bZIP members from potato (A) and Arabidopsis (B). [file Data_Sheet_1.ZIP › supplementary materials/Supplementary Figure S1.pdf]

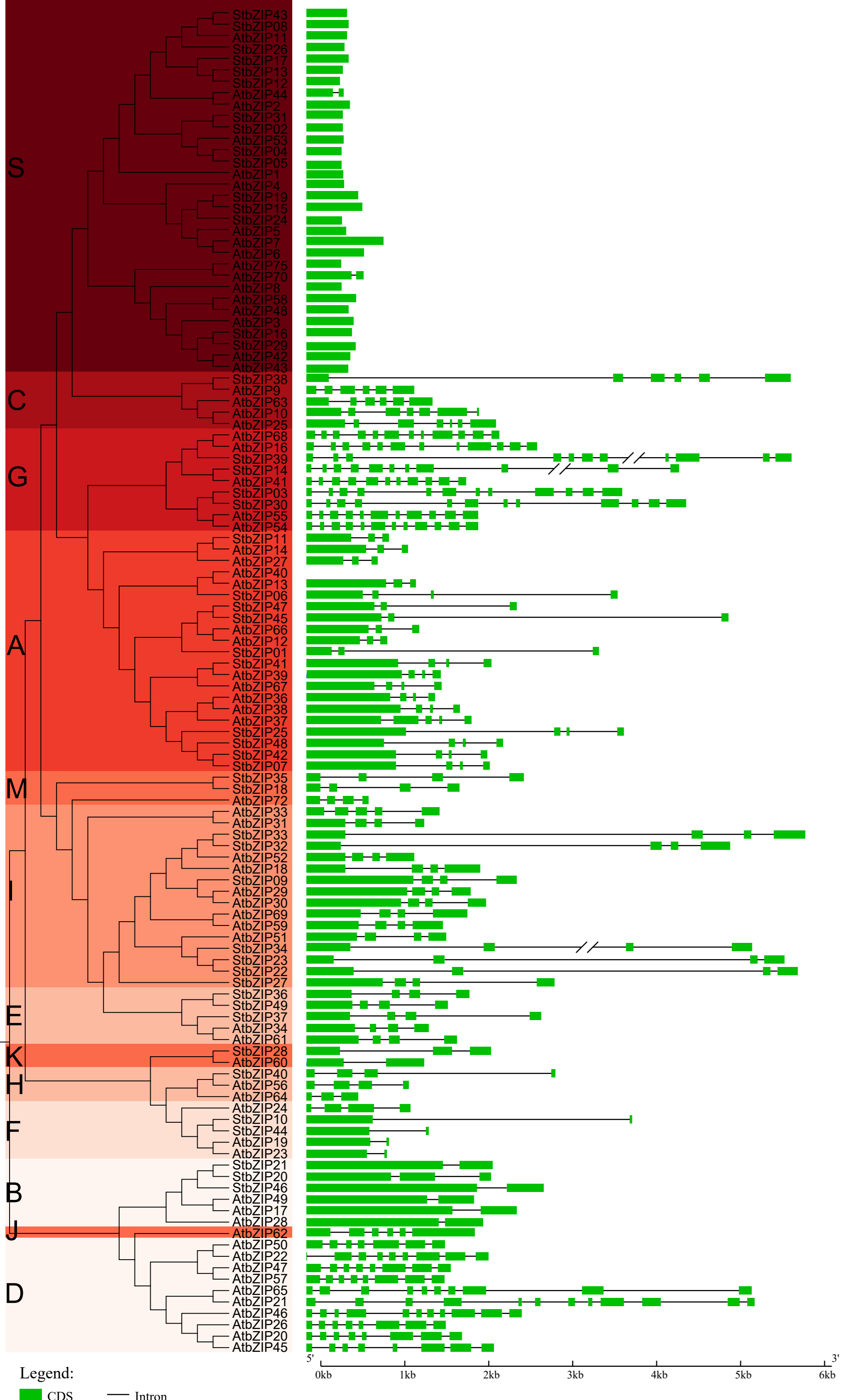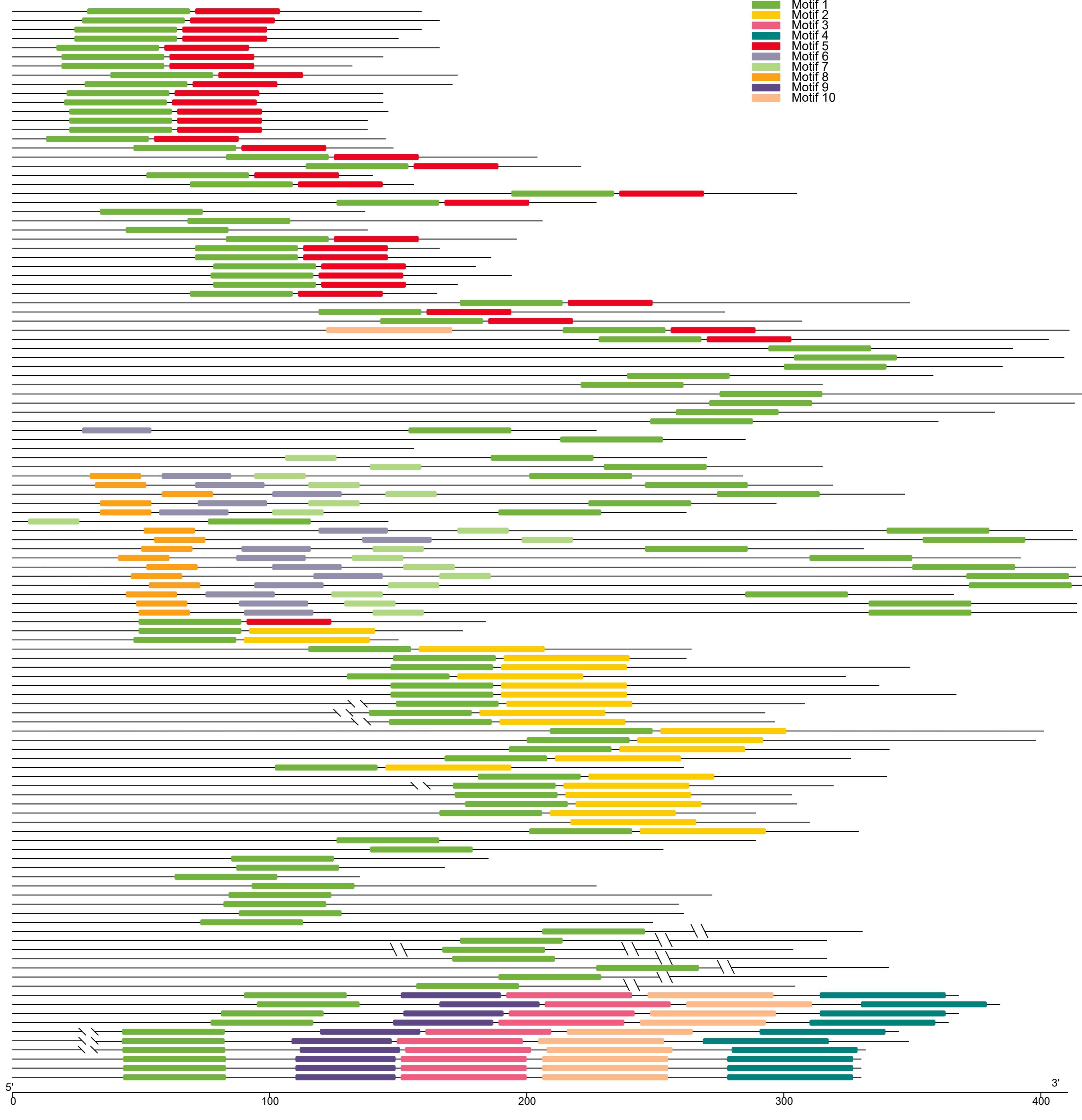

Supplement: Supplementary Figure 1 — Sequence logos of the basic region of bZIP members from potato (A) and Arabidopsis (B). [file Data_Sheet_1.ZIP › supplementary materials/Supplementary Figure S2.pdf]

A

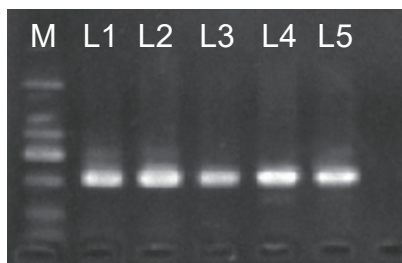

B

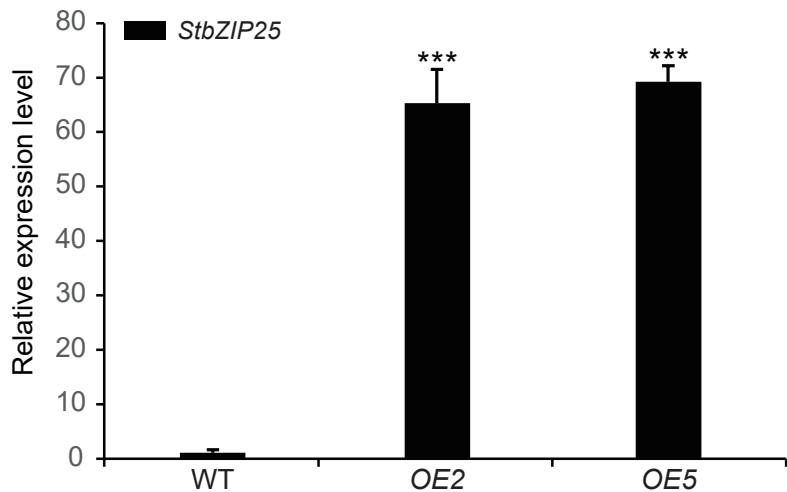

Supplement: Supplementary Figure 1 — Sequence logos of the basic region of bZIP members from potato (A) and Arabidopsis (B). [file Data_Sheet_1.ZIP › supplementary materials/Supplementary Figure S3.pdf]

Motif1


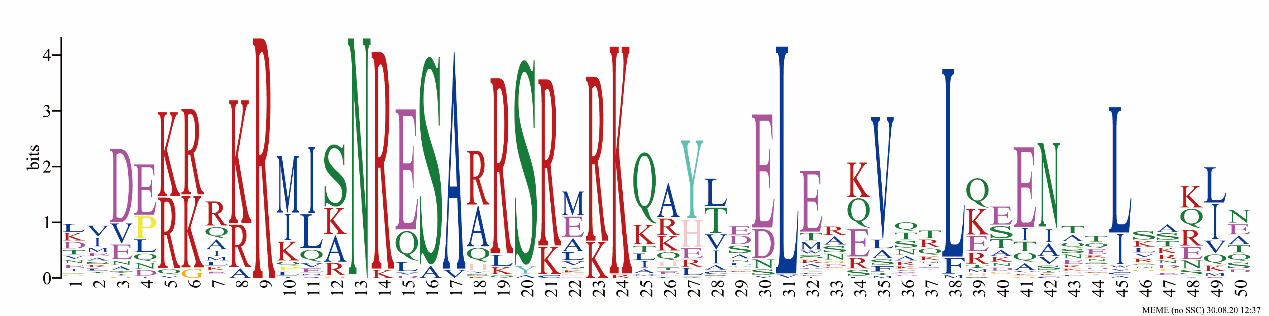


Motif2


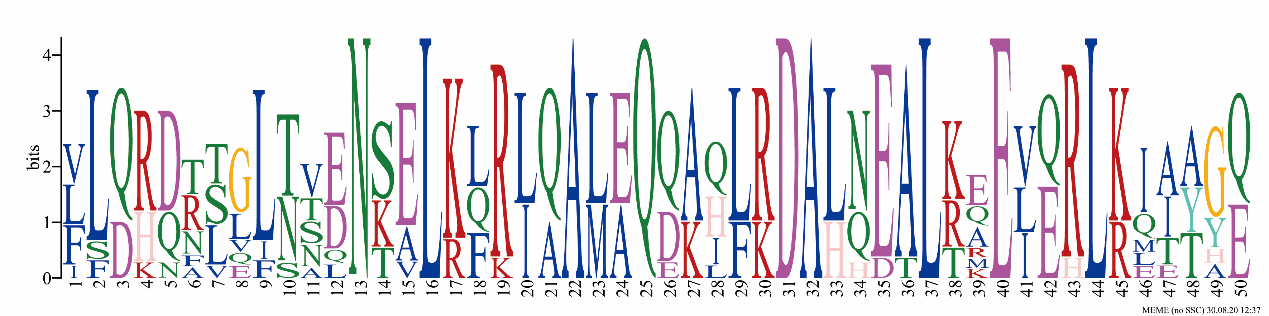


Motif3


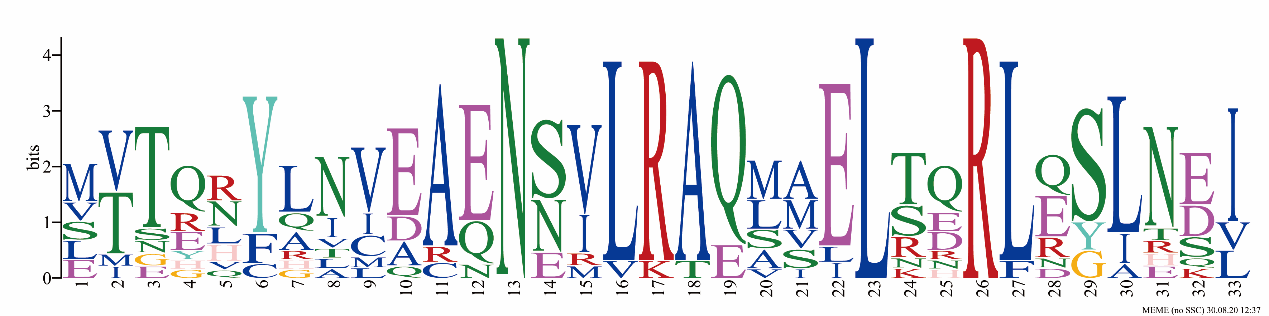


Motif4


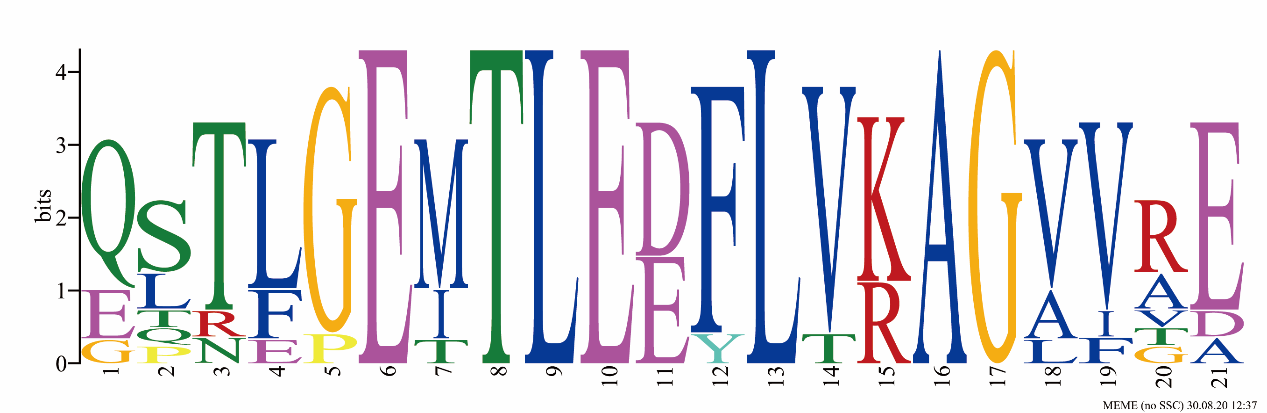


Motif5


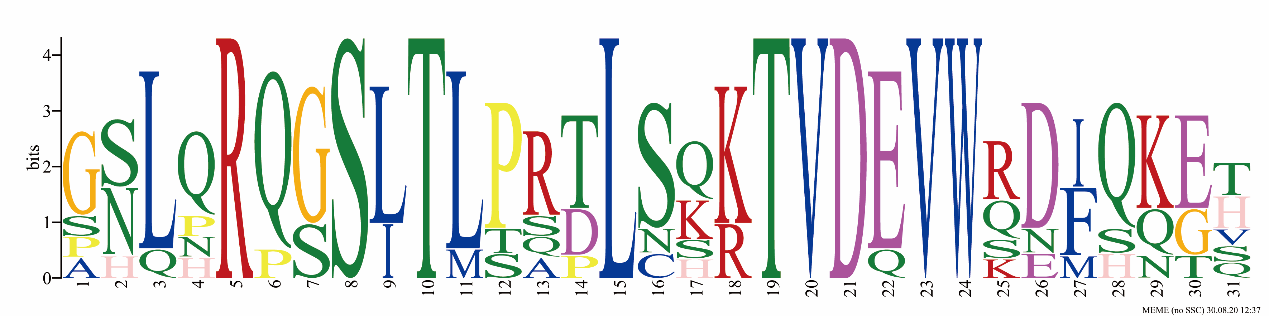


Motif6


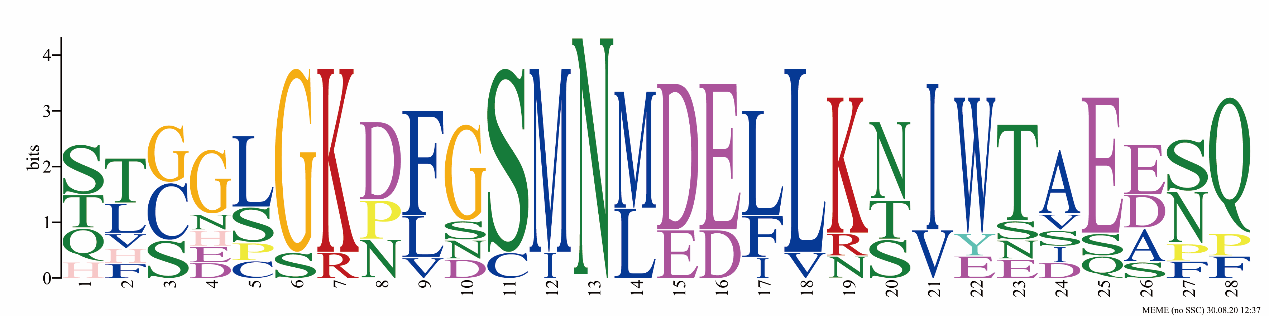


Motif7


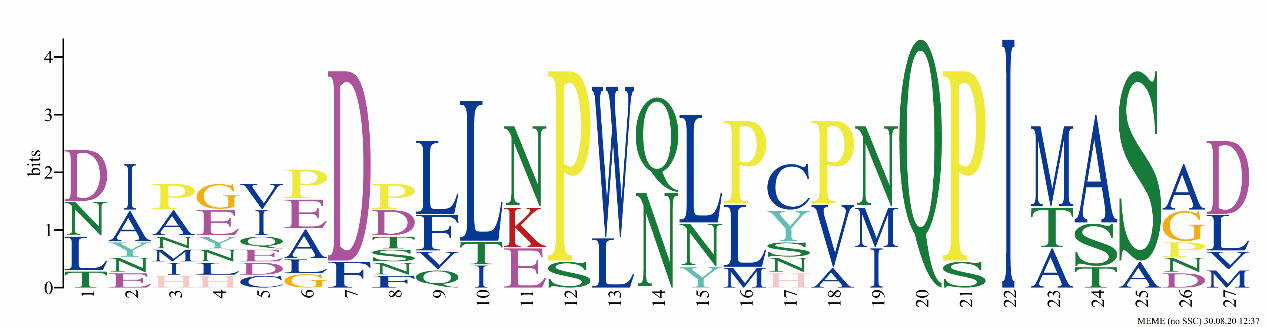


Motif8


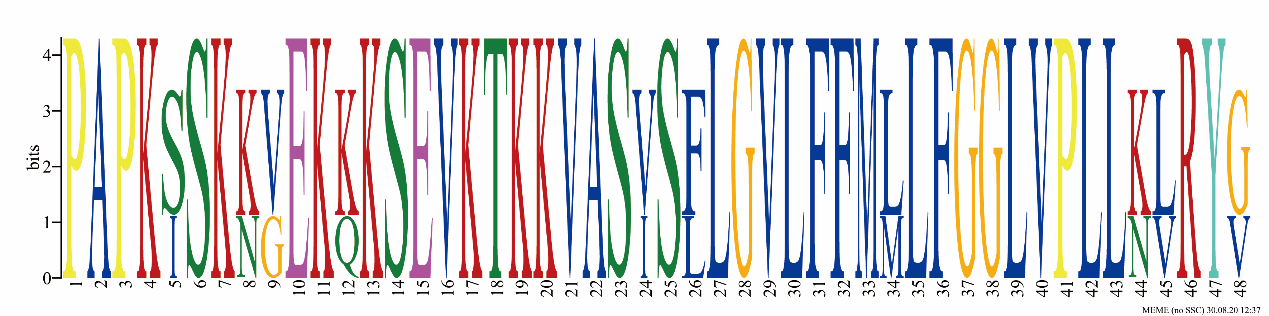


Motif9


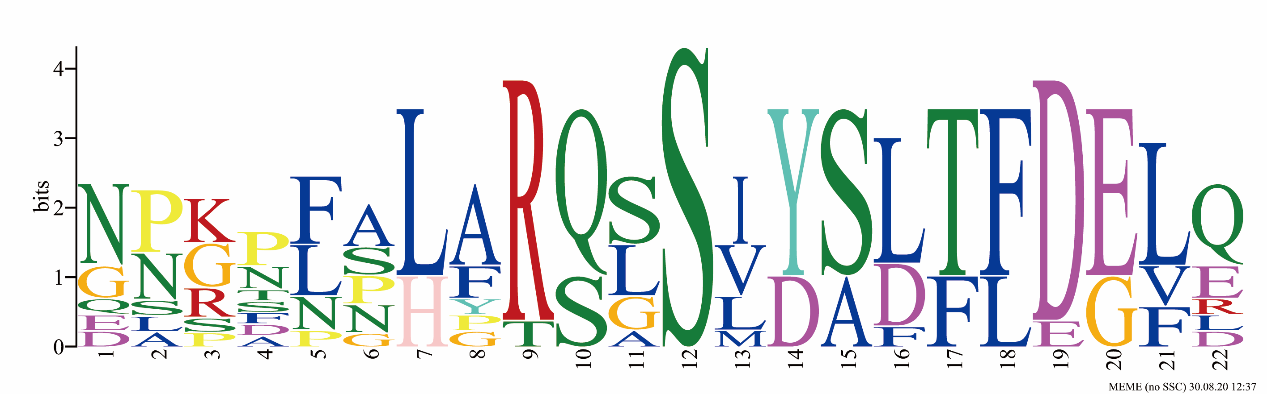


Motif10


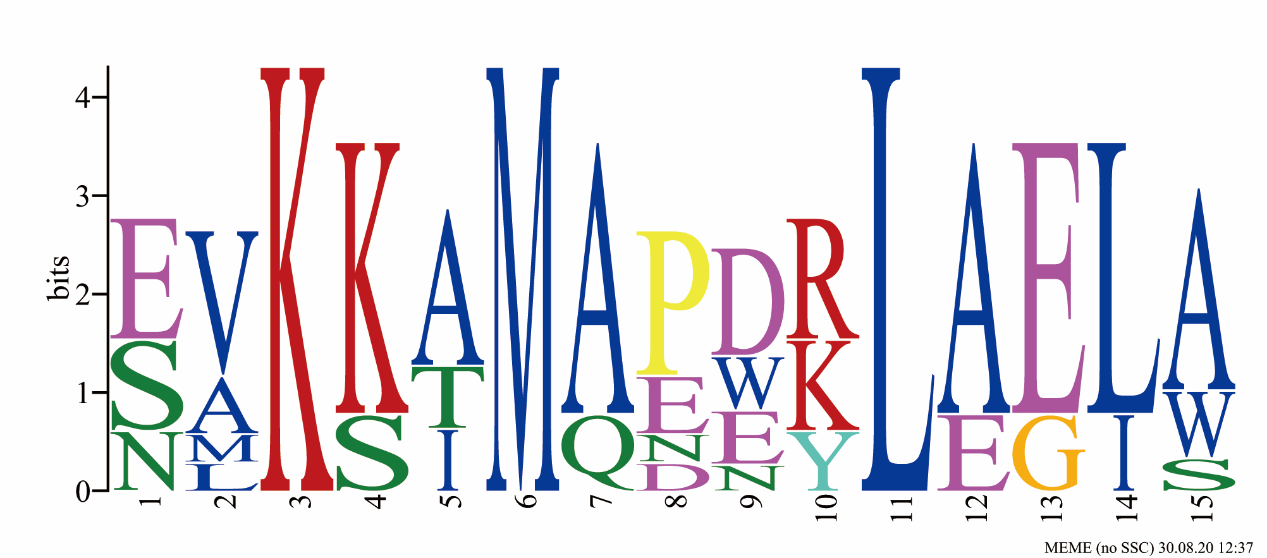

Supplement: Supplementary Figure 1 — Sequence logos of the basic region of bZIP members from potato (A) and Arabidopsis (B). [file Data_Sheet_1.ZIP › supplementary materials/Supplementary Table S4.docx]
